# Intramembranous Bone Healing Process Subsequent to Tooth Extraction in Mice: Micro-Computed Tomography, Histomorphometric and Molecular Characterization

**DOI:** 10.1371/journal.pone.0128021

**Published:** 2015-05-29

**Authors:** Andreia Espindola Vieira, Carlos Eduardo Repeke, Samuel de Barros Ferreira Junior, Priscila Maria Colavite, Claudia Cristina Biguetti, Rodrigo Cardoso Oliveira, Gerson Francisco Assis, Rumio Taga, Ana Paula Favaro Trombone, Gustavo Pompermaier Garlet

**Affiliations:** 1 Department of Biological Sciences, Bauru School of Dentistry, University of São Paulo, Bauru, SP, Brazil; 2 Department of Biological and Allied Health Sciences, Sacred Heart University, Bauru, Brazil; University of Ulm, GERMANY

## Abstract

Bone tissue has a significant potential for healing, which involves a significant the interplay between bone and immune cells. While fracture healing represents a useful model to investigate endochondral bone healing, intramembranous bone healing models are yet to be developed and characterized. In this study, a micro-computed tomography, histomorphometric and molecular (RealTimePCRarray) characterization of post tooth-extraction alveolar bone healing was performed on C57Bl/6 WT mice. After the initial clot dominance (0h), the development of a provisional immature granulation tissue is evident (7d), characterized by marked cell proliferation, angiogenesis and inflammatory cells infiltration; associated with peaks of growth factors (BMP-2-4-7,TGFβ1,VEGFa), cytokines (TNFα, IL-10), chemokines & receptors (CXCL12, CCL25, CCR5, CXCR4), matrix (Col1a1-2, ITGA4, VTN, MMP1a) and MSCs (CD105, CD106, OCT4, NANOG, CD34, CD146) markers expression. Granulation tissue is sequentially replaced by more mature connective tissue (14d), characterized by inflammatory infiltrate reduction along the increased bone formation, marked expression of matrix remodeling enzymes (MMP-2-9), bone formation/maturation (RUNX2, ALP, DMP1, PHEX, SOST) markers, and chemokines & receptors associated with healing (CCL2, CCL17, CCR2). No evidences of cartilage cells or tissue were observed, strengthening the intramembranous nature of bone healing. Bone microarchitecture analysis supports the evolving healing, with total tissue and bone volumes as trabecular number and thickness showing a progressive increase over time. The extraction socket healing process is considered complete (21d) when the dental socket is filled by trabeculae bone with well-defined medullary canals; it being the expression of mature bone markers prevalent at this period. Our data confirms the intramembranous bone healing nature of the model used, revealing parallels between the gene expression profile and the histomorphometric events and the potential participation of MCSs and immune cells in the healing process, supporting the forthcoming application of the model for the better understanding of the bone healing process.

## Introduction

Bone is comprised of complex mineralized connective tissue characterized by constant remodeling, which involves cycles of bone resorption coupled with subsequent bone formation [1]. Bone tissue also has considerable potential for healing, which involves the cooperative action of bone forming and resorptive cells to restore the architecture and function of damaged tissue [2, 3]. The bone healing process is triggered by injury, which results in a local inflammatory immune reaction whose development is thought to highly influence the outcome of the bone healing process [4, 5]. In fact, inflammatory immune responses have been described in playing a significant role in bone healing, as originally demonstrated by the association between anti-inflammatory drugs and delayed bone healing [6, 7].

However, after the early descriptions of the inflammation-bone healing association in the late 70´s/early 80´s, the knowledge related to the interplay between bone and the immune systems presented relatively small advances until the discovery of the RANK/RANKL/OPG system, composed by molecules shared by both systems, which ultimately resulted in the birth of the terminology ‘osteoimmunology’ [8]. However, despite the primary evidences linking bone and immunological systems within the bone healing process, most of the studies in the field of osteoimmunology are focused on chronic inflammatory osteolytic conditions, being the putative influence of immune inflammatory mediators in the healing process poorly explored [7, 9–11]. The scarce knowledge related to the osteoimmunological aspects of bone healing is essentially derived from fracture healing studies [7, 10, 12]. In a general context, such studies confirm the requirement of a transitory and moderate inflammatory process for proper bone healing, leading to the concept of constructive inflammation [13–15]. In this scenario, the inflammatory events could contribute to the local production/release of growth factors classically associated with bone neoformation (such as BMPs and TGF-β), as well with the promotion of chemotaxis of cells associated with the repair process [2, 16–19]. However, even in the fracture healing scenario, the exact mechanisms coupling the host inflammatory immune response with the bone healing process remains to be determined. In addition, due to the peculiarities of bone tissue present in different areas of the vertebrates’ endoskeleton, knowledge of the fracture healing process cannot be integrally extrapolated to the overall context of bone healing.

Among the specialized bone tissues, the alveolar bone supporting the tooth in the maxilla and mandible is characterized by distinctive features such as the continuous and rapid remodeling in response to stimuli by force [20, 21]. Alveolar bone finds itself in an unceasing adaptation to functional demands, such as the forces derived from mastication and swallowing, and in the absence of such stimuli, the alveolar bone undergoes a resorptive process [21, 22]. Another particularity that comprises the different nature of cells surrounding the alveolar and ‘regular’ (i.e. non-alveolar) bone are the muscle stem cells, absent in alveolar bone, which plays a critical role in fracture healing [23–26]. While long bone healing occurs by endochondral ossification, alveolar bone healing typically occurs without histological cartilage formation [27]. Also, opposite the bone fracture sites, usually considered a sterile milieu, oral tissues surrounding the alveolar bone are under a constant microbial challenge. In fact, infectious processes are characteristically associated with impaired bone healing in the oral cavity [28–30].

Therefore, while fracture healing models have proven to be useful tools to investigate endochondral bone healing [7, 10, 12], the development and characterization of an alveolar bone healing model is extremely important to provide an instrument for further specific investigations. Several clinical procedures, such as implant-based rehabilitative therapies, depend on the proper healing of alveolar bone, and would greatly benefit from a better understanding of this process, and the consequent improvement of alveolar bone regenerative strategies [3]. In this context, alveolar bone healing following mice tooth extractions can present an interesting and useful bone healing experimental model. Considering the emerging specific interest in the interplay between the immune and bone systems, the use of mice as an experimental model hosts result in additional advantages due the extensive knowledge regarding mice inflammatory and immunological responses, as well as the existence of multiple strains (including numerous genetically modified ones), which allow for a broad variety of experimental opportunities.

Therefore, in this study, we performed the microtomographic, histological, histomorphometric and molecular characterization of the alveolar bone healing process in mice following tooth extractions, to fully characterize the intramembranous bone healing process and support its future application in the field.

## Materials and Methods

### Animals

The experimental groups were comprised of 8-week-old male wild-type (WT) C57BL/6 mice, bred in the animal facilities of USP. Throughout the study period, sterile water *ad libitum* was provided and the mice were fed with sterile standard solid mice chow (Nuvital, Curitiba, PR, Brazil), except in the first 24 hours after surgery, in which the diet was crumbled. The experimental groups were comprised of 9 mice (5 for both the microtomographic [μCT], histological and histochemical analysis, and 4 for the RealTimePCRarray analysis).

### Ethics Statement

This study was carried out in strict accordance with the recommendations in the Guide for the Care and Use of Laboratory Animals of the National Institutes of Health. The experimental protocol was approved by the local Institutional Committee for Animal Care and Use (Committee on Animal Research and Ethics [Comissão de Ética no Ensino e Pesquisa em Animais] CEEPA-FOB/USP, processes #026/2009 & #029/2009). The animals were anaesthetized by intramuscular administration of 80mg/kg of ketamine chloride (Dopalen, Agribrans Brasil LTDA) and 160mg/kg of xylazine chloride (Anasedan, Agribrands Brasil LTDA) in the proportion 1:1 determined according to the animal body mass. At the end of the experimental periods (0h, 7, 14 and 21 days post tooth extraction), the animals were killed with an excessive dose of anesthetic.

### Experimental protocol and mice tooth extraction model

The animals were anaesthetized by intramuscular administration of 80mg/kg of ketamine chloride (Dopalen, Agribrans Brasil LTDA) and 160mg/kg of xylazine chloride (Anasedan, Agribrands Brasil LTDA) in the proportion 1:1, determined according to the animal body mass. The extraction of the upper right incisor was performed with the aid of a stereomicroscope (DF Vasconcellos S.A., Sao Paulo, Brasil) under 25x magnification. A dental exploratory probe was used to promote the dental element luxation by a smooth movement to avoid root fracture, followed by the use of clinical tweezers to seize and remove the tooth (S1 Fig). After extraction, the removed tooth was checked for integrity. Animals presenting fractured teeth during the extraction were excluded from further analysis. At the end of the experimental periods (0h, 7, 14 and 21 days post tooth extraction), the animals were killed with an excessive dose of anesthetic, and the maxillae were collected. Five maxillae were destined for micro-computed tomography (μCT), histological and histochemical analyses; and four samples containing only the region of the alveolus were destined for the RealTimePCRarray analysis. Samples for the μCT and histological analyses were fixed in PBS-buffered formalin (10%) solution (pH 7.4) for 48h at room temperature, subsequently washed over-night in running water and maintained temporarily in alcohol fixative (70% hydrous ethanol) until the conclusion of the μCT analysis [31], and them decalcified in 4.13% EDTA (pH 7,2) and submitted to histological processing. Samples for molecular analysis were stored in RNAlater (Ambion, Austin, TX) solutions [32, 33].

### Micro-computed tomography (μCT) assessment

The maxillae samples were scanned by the Skyscan 1174 System (Skyscan, Kontich, Belgium) at 50 kV, 800 μA, with a 0.5 mm aluminium filter and 15% beam hardening correction, ring artifacts reduction, 180 degrees of rotation and exposure range of 1 degree. Images were captured with 1304x1024 pixels and a resolution of 14μm pixel size. Projection images were reconstructed using the NRecon software and three-dimensional images obtained by the CT-Vox software. Morphological parameters of trabecular bone microarchitecture were assessed using the CTAn software in accordance with the recommended guidelines [34]. A cylindrical region of interest (ROI) with an axis length of 3mm (100 slices) and diameter of 1mm was determined by segmenting the trabecular bone located from the coronal to apical thirds. Trabecular measurements analyzed included the tissue volume (TV), bone volume (BV) bone volume fraction (BV/TV, %), trabecular thickness (Tb.Th, mm), trabecular number (Tb.N, mm), and trabecular separation (Tb.Sp) [34, 35].

### Histomorphometric analysis

Serial sections (8 semi-serial sections of each maxilla, with a 5 μm thickness for each section) were obtained using a microtome (Leica RM2255, Germany) and stained with H.E. (hematoxylin and eosin). Morphometric measurements were performed by a single calibrated investigator with a binocular light microscope (Olympus Optical Co., Tokyo, Japan) using a 100x immersion objective and a Zeiss kpl 8X eyepiece containing a Zeiss II integration grid (Carl Zeiss Jena GmbH, Jena, Germany) with 10 parallel lines and 100 points in a quadrangular area. The grid image was successively superimposed on approximately 13 histological fields per histological section, comprised of all tooth sockets from the coronal limit adjacent to the gingival epithelium until the lower apical limit. For each animal/socket, sections from the coronal, medial and apical thirds were evaluated. In the morphometric analysis, points were counted coinciding with the images of the following components of the alveolar socket: clot, inflammatory cells, blood vessels, fibroblasts, collagen fibers, bone matrix, osteoblasts, osteoclasts and other components (empty space left by the inflammatory exudate or intercellular liquid and bone marrow); similar to previous descriptions [5, 36–38]. The results are presented as the volume density (mean) for each evaluated structure.

### Picrosirius-polarization method and quantification of birefringent fibers

The Picrosirius-polarization method and quantification of birefringent fibers were performed to assess the structural changes in the newly formed bone trabeculae matrix based on the birefringence of the collagen fiber bundles, as previously described [39, 40]. Serial sections (8 semi-serial sections of each maxilla) with 5 μm thickness were cut and stained with Picrosirius Red Stain; all sections were stained simultaneously to avoid variations due to possible differences in the staining process. Picrosirius Red-stained sections were analyzed through a polarizing lens coupled to a binocular inverted microscope (Leica DM IRB/E), and all images were captured with the same parameters (the same light intensity and angle of the polarizing lens 90° to the light source). AdobePhotoshopCS6 software was used to delimit the region of interest (alveolar area comprised of new tissue with the external limit comprised of the alveolar wall), totalizing 1447680 pixels^2^. The quantification of the intensity of birefringence brightness was performed using the AxioVision 4.8 software (CarlZeiss). For quantification, the images were binarized for definition of the green, yellow and red color spectra, and the quantity of each color pixels^2^ corresponding to the total area enclosed in the alveoli were measured. Mean values of 4 sections from each animal were calculated in pixels^2^.

### RealTimePCR array reactions

RealTimePCR array reactions were performed as previously described [41–43]. The extraction of total RNA from the remaining alveolus was performed with the RNeasyFFPE kit (Qiagen Inc, Valencia, CA) according to the manufacturers’ instructions. The integrity of the RNA samples was verified by analyzing 1 mg of total RNA in a 2100Bioanalyzer (Agilent Technologies, Santa Clara, CA) according to the manufacturers’ instructions, and the complementary DNA was synthesized using 3 μg of RNA through a reverse transcription reaction (Superscript III, Invitrogen Corporation, Carlsbad, CA, USA). RealTimePCR array was performed in a Viia7 instrument (LifeTechnologies, Carlsbad, CA) using a custom panel containing targets "Wound Healing" (PAMM-121), "Inflammatory cytokines and receptors"(PAMM-011) and "Osteogenesis" (PAMM-026) (SABiosciences, Frederick, MD) for gene expression profiling. RealTimePCR array data was analyzed by the RT2 profiler PCR Array Data Analysis online software (SABiosciences, Frederick, MD) for normalizing the initial geometric mean of three constitutive genes (GAPDH, ACTB, Hprt1) and subsequently normalized by the control group, and expressed as fold change relative to the control group; as previously described [44, 45].

### Statistical analysis

Differences among data sets were statistically analyzed by One-Way analysis of variance (ANOVA) followed by the Bonferroni's multiple comparison posttest or the student's t-test where applicable; for data that did not fit in the distribution of normality, the Mann-Whitney and Kruskal-Wallis (followed by the Dunn's test) tests were used. The statistical significance of the experiment involving the PCR Array was evaluated by the Mann-Whitney test, and the values tested for correction by the Benjamini—Hochberg Procedure [46]. Values of p<0.05 were considered statistically significant. All statistical tests were performed with the GraphPad Prism 5.0 software (GraphPad Software Inc., San Diego, CA, USA).

## Results

### μCT analysis

**Fig 1:**
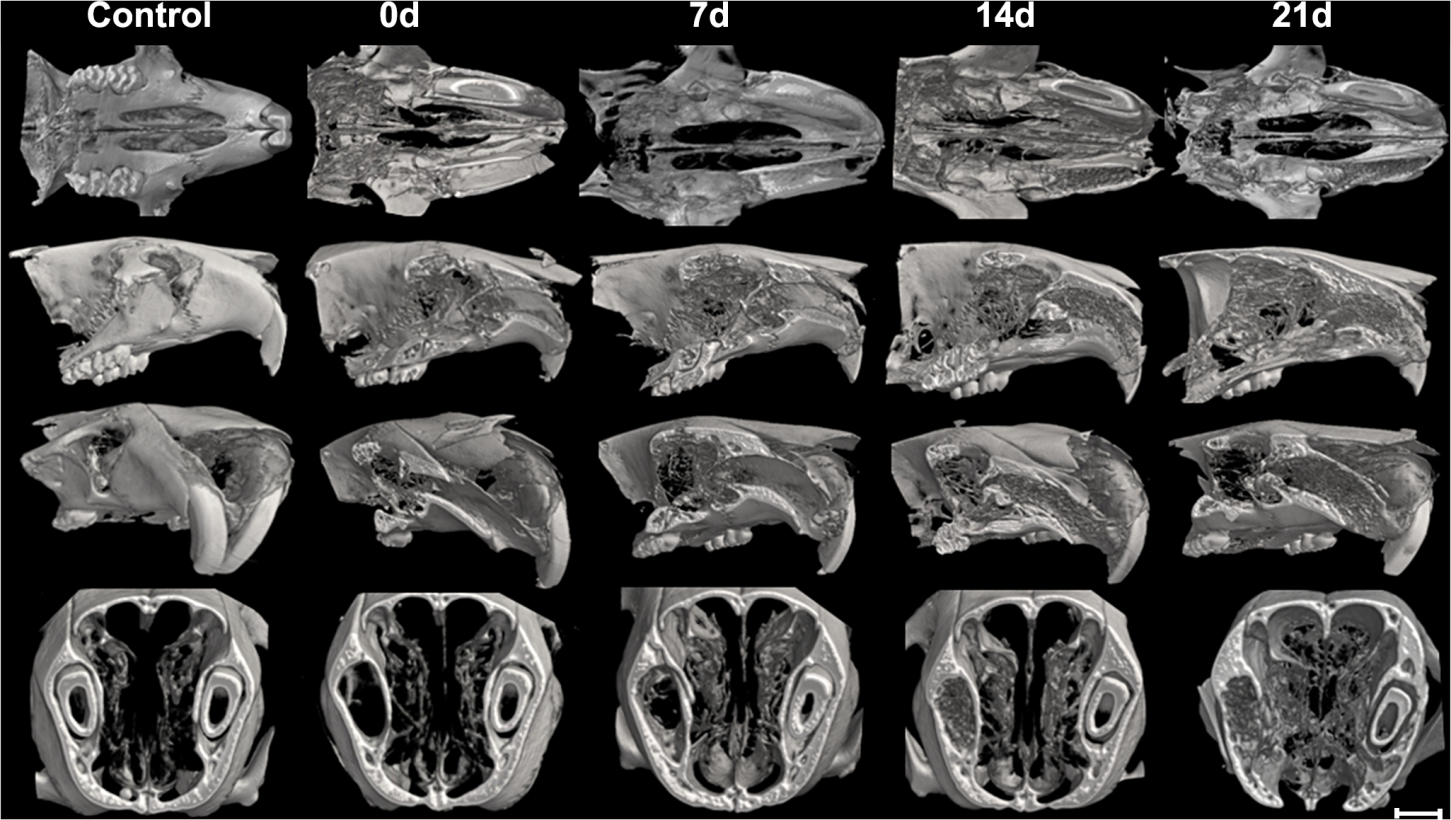
Micro-computed tomography (μCT) analysis of bone healing process kinetics in mice. Samples from 8-week-old male wild-type (WT) C57BL/6 mice were scanned with the μCT System (Skyscan 1174; Skyscan, Kontich, Belgium): control (maxilla without extraction) and at 0, 7, 14 and 21 days post tooth to evaluate the kinetics of the bone healing process. Images were reconstructed using the NRecon software and three-dimensional images obtained with the CT-Vox software. From top to bottom, the sectioned maxilla are represented at the transverse (horizontal); sagittal; sagittal with inclination and transaxial planes.

Three-dimensional images from the μCT of maxillae containing alveolar bone healing revealed the extraction sockets at the initial period (0 hour time point) completely void with lack of hyperdense areas (Fig 1), while at 7 days, hyperdense areas were evidenced compatible with the beginning of bone formation centripetal from the lateral and apical walls of the extraction sockets toward the center and the coronal region of the alveolus. At 14 days, the hyperdense areas evidenced more advanced bone formation with the trabecular bone reaching the central region of the socket, while at 21 days, larger amounts of hyperdense areas with thicker bone trabeculae are observed along the entire length of the socket, evidencing that the extraction sockets were successfully healed (Fig 1). The morphological observations derived from the μCT reconstruction were confirmed when the bone microarchitecture features were quantitatively evaluated (Table 1). Total tissue volume (TV), bone volume (BV), and bone volume fraction (BV/TV) progressively increased over the periods, as well as the trabecular thickness (Tb.Th). Furthermore, there was a marked increase in trabecular number (Tb.N) and a reduction in trabecular separation (Tb.Sp) from 14 days (Table 1).

**Table 1:** Trabecular bone microarchitecture from μCT assessment of the tooth extraction sockets along the bone healing process.

Parameters	0d	7d	14d	21d
TV (mm^3^)	0.81±0.017^a^	1.79±0.43^b^	1.96±0.07^b^	2.15±0.11^b^
BV (mm^3^)	0.02±0,00^a^	0,17±0.05^a^	1.07±0.16^b^	1.33±0.08^c^
BV/TV (%)	1.41±0.38^a^	9.59±1.71^a^	54.64±7.62^b^	61.97±1.99^b^
Tb.Th (mm)	0.00±0.00^a^	0.06±0.01^b^	0.15±0.01^c^	0.19±0.02^c^
Tb.N (1/mm)	0.00±0.00^a^	1.46±0.21^b^	3.51±0.20^c^	3.23±0.36^c^
Tb.Sp (mm)	0.00±0.00^a^	0.51±0.05^b^	0.13±0.01^c^	0.13±0.01^d^

Morphological parameters of the trabecular bone microarchitecture were assessed using the CTAn software from the cylindrical region of interest (ROI) determined by segmenting the trabecular bone located from the coronal to apical thirds. Trabecular measurements analyzed included the tissue volume (TV), bone volume (BV) bone volume fraction (BV/TV, %), trabecular thickness (Tb.Th, mm), trabecular number (Tb.N, mm), and trabecular separation (Tb.Sp). Different letters indicate significant statistical differences (p 0.05) between time periods.

### Histological, histomorphometric, immunohistochemistry and matrix birefringence analyses

**Fig 2:**
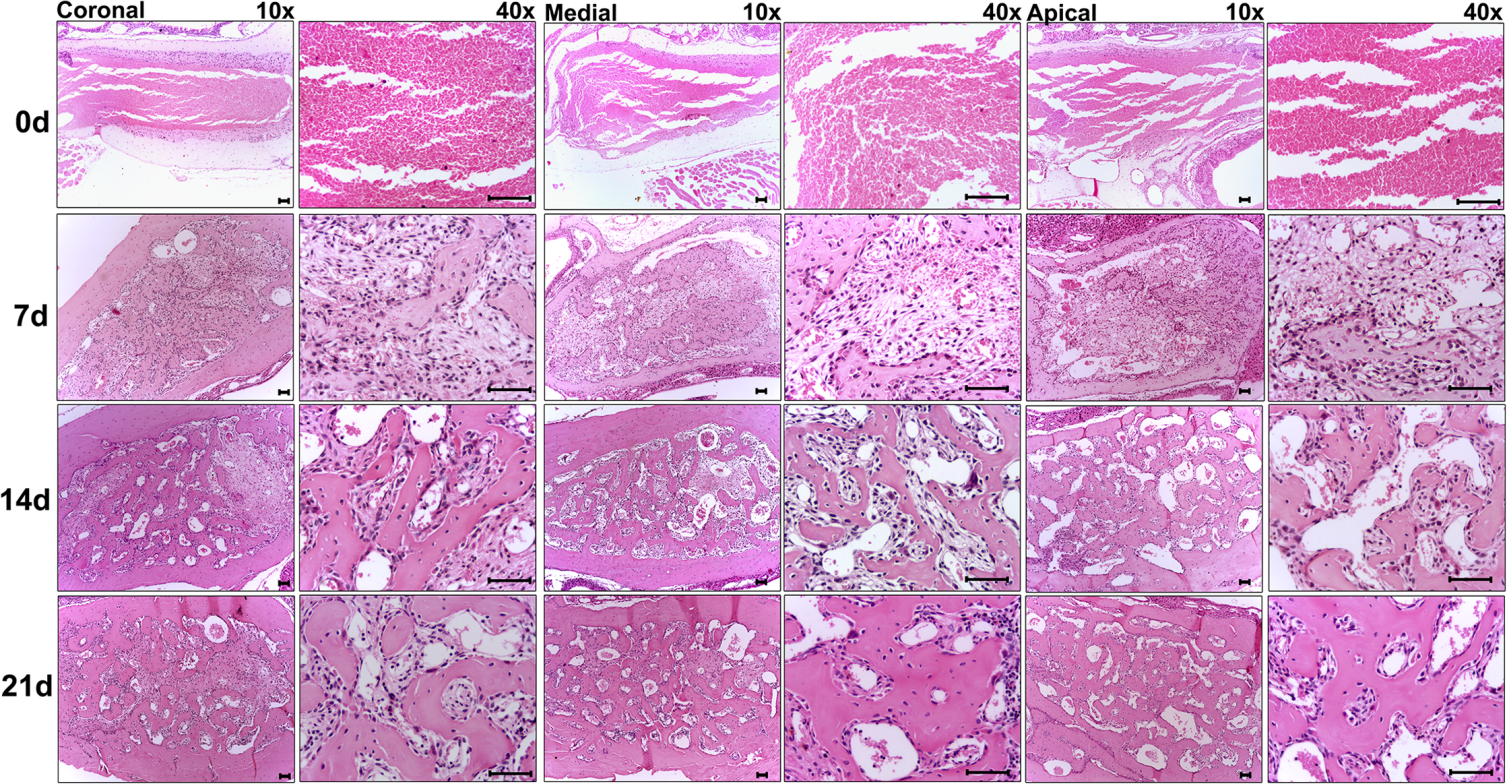
Histological aspects of the coronal, medial and apical thirds from tooth sockets in the bone healing process. Representative sections of the alveolar bone healing kinetics at 0, 7, 14 and 21 days post-extraction of the upper right incisor. HE staining, original magnification 10x and 40x. Bar = 100 μm.

**Fig 3:**
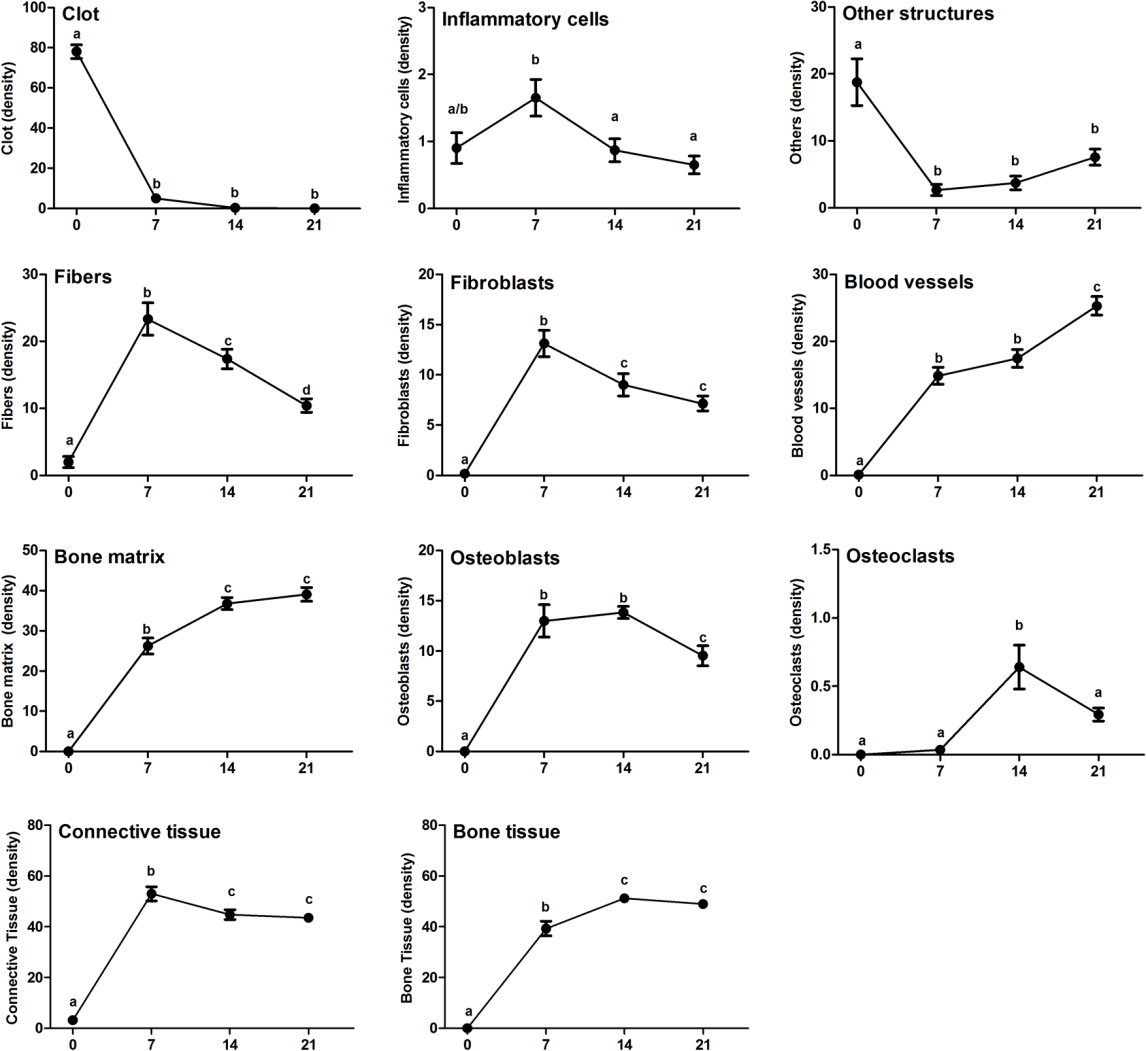
Histomorphometric analysis of alveolar bone healing kinetics after tooth extraction. Results are presented as the means (±SEM) of density for each structure of the alveolar socket: clot, inflammatory cells, blood vessels, fibroblasts, collagen fibers, bone matrix, osteoblasts, osteoclasts and other components (empty space left by the inflammatory exudate or intercellular liquid and bone marrow). Also, the results depict the total density of connective tissue (represented by the sum of inflammatory cells, collagen fibers, fibroblasts and blood vessels) and bone tissue (represented by the sum of its structural components bone matrix, osteoblasts and osteoclasts). Different letters indicate a statistically significant difference (p 0.05) between the different time periods (p<0.05).

**Fig 4:**
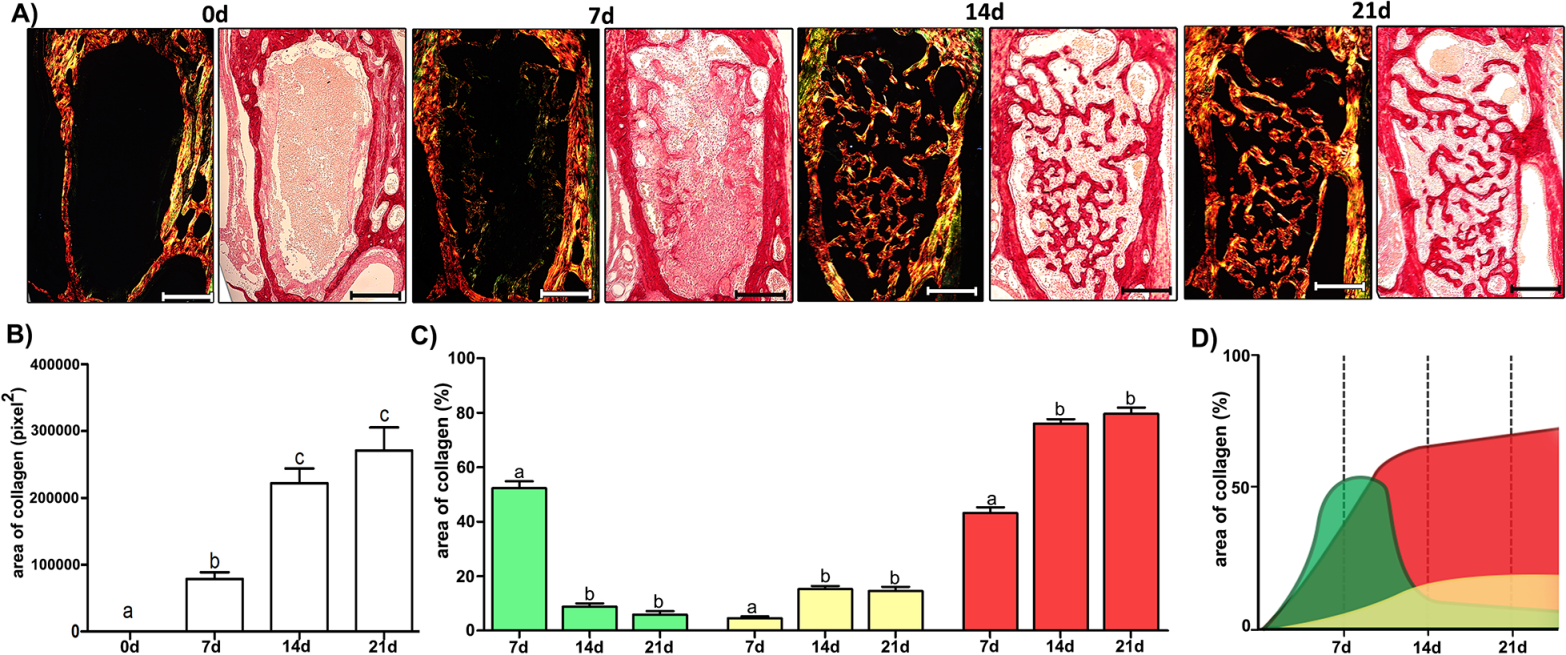
Quantification of birefringent fibers by the picrosirius-polarization method in the bone healing process after tooth extraction. (A) Representative sections of Picrosirius red staining visualized upon polarized and conventional light to identify collagen fibers types at 0 hour, 7, 14 and 21 days post-extraction of the upper right incisor. Green birefringence color indicates thin fibers; yellow and red colors in the birefringence analysis indicate thick collagen fibers. Original magnification 10x. Bar = 100 μm. Intensity of birefringence was measured with the Image-analysis software (AxioVision, v. 4.8, CarlZeiss) to identify and quantify: (B) total area of collagen fibers (pixel^2^) and (C) area of collagen from each birefringence color (%). Results are presented as the mean (±SEM) of pixels^2^ for each color in the birefringence analysis in the bone healing at 0 hour, 7, 14 and 21 days post-extraction. (D) Graphic representation of area of collagen fibers (%) maturation dynamics in the bone healing of the socket.

The histological and histomorphometric analyses demonstrated that the healing process exhibited sequential and at some extent overlapping phases characterized by the presence of clot; migration and proliferation of MSCs; granulation tissue formation with inflammatory cells infiltration, angiogenesis, proliferation of fibroblasts and collagen synthesis; bone formation and remodeling (Fig 2); such descriptive analysis is supported by the histomorphometric analysis of each parameter (Fig 3). At baseline 0h time period, immediately after tooth extraction, the socket was occupied by a blood clot in its full extension, along with the presence of leukocytes and empty spaces, possibly comprised of the inflammatory exudate. While the clot density reduced significantly between the 0h and 7d time periods (p< 0.05 vs. 0h), the inflammatory infiltrate presented an increase at 7d, followed by a significant and gradual reduction in the subsequent time periods (p>0,05; 7d vs 14d). At 7d, the presence of granulation tissue was characterized by numerous newly formed small blood vessels, an intense inflammatory cell infiltrate and immature connective tissue with large amounts of fibroblasts and immature collagen fiber bundles (Fig 4). While at the 0h baseline time period, birefringent fibers were not detected inside the socket, at the 7d time period, fiber emitting birefringence in green tones (i.e immature fibers) (Fig 4) were found in profusion. Blood vessel volume density in the alveolar sockets showed a significant increase at the 7d time period (p< 0.05 vs. 0h), followed by an additional increase at 14d and 21d (p<0,05 vs 0h). A significant increase in the fibroblast and collagen fibers volume density at 7d (p<0,05 vs 0h) was followed by a reduction in the subsequent periods (14d and 21d). This data is consistent with the results from the immunohistochemistry targeting PCNA (proliferating cell nuclear antigen), which demonstrated a high labeling of cell proliferation at 7 days that gradually decreased (S2 Fig). At 14 days, the maturation of connective tissue was evidenced by the presence of mature collagen fiber bundles (Fig 4), when about three quarters of the fibers presented the red color spectrum, representative of a mature matrix (Fig 4). A sequential decrease in the density of non-mineralized connective tissue, fibroblasts and inflammatory cell volume density (p< 0.05), parallel with new bone formation was also observed (Fig 3). At the 7d time period, newly formed woven primary bone (immature) with numerous newly differentiated osteoblasts and developing bone matrix with a high proportion of osteocytes appeared on the lining of the socket walls and expanding to the central region of the socket with a centripetal pattern, leading to the confluence of bone trabeculae (Fig 2). Accordingly, the osteoblasts volume density revealed a significant increase at 7d and 14d (p< 0.05 vs. 0h), with a posterior reduction at the 21d time period (p> 0.05 vs. 0h). Osteoclasts courting peaked at 14d (p< 0.05), and were also observed in small numbers at the different periods evaluated. Finally, the histomorphometric analysis showed a higher quantified volume density than the other structures in the initial alveolar healing process, probably due the presence of interstitial fluid, and the late periods, represented by bone marrow (Figs 2 and 3). The proportion and density of all the analyzed structures did not present statistically significant differences when the results from the coronal, middle and apical thirds of the sockets were analyzed individually (data not shown).

### Gene expression patterns in the bone healing

**Fig 5:**
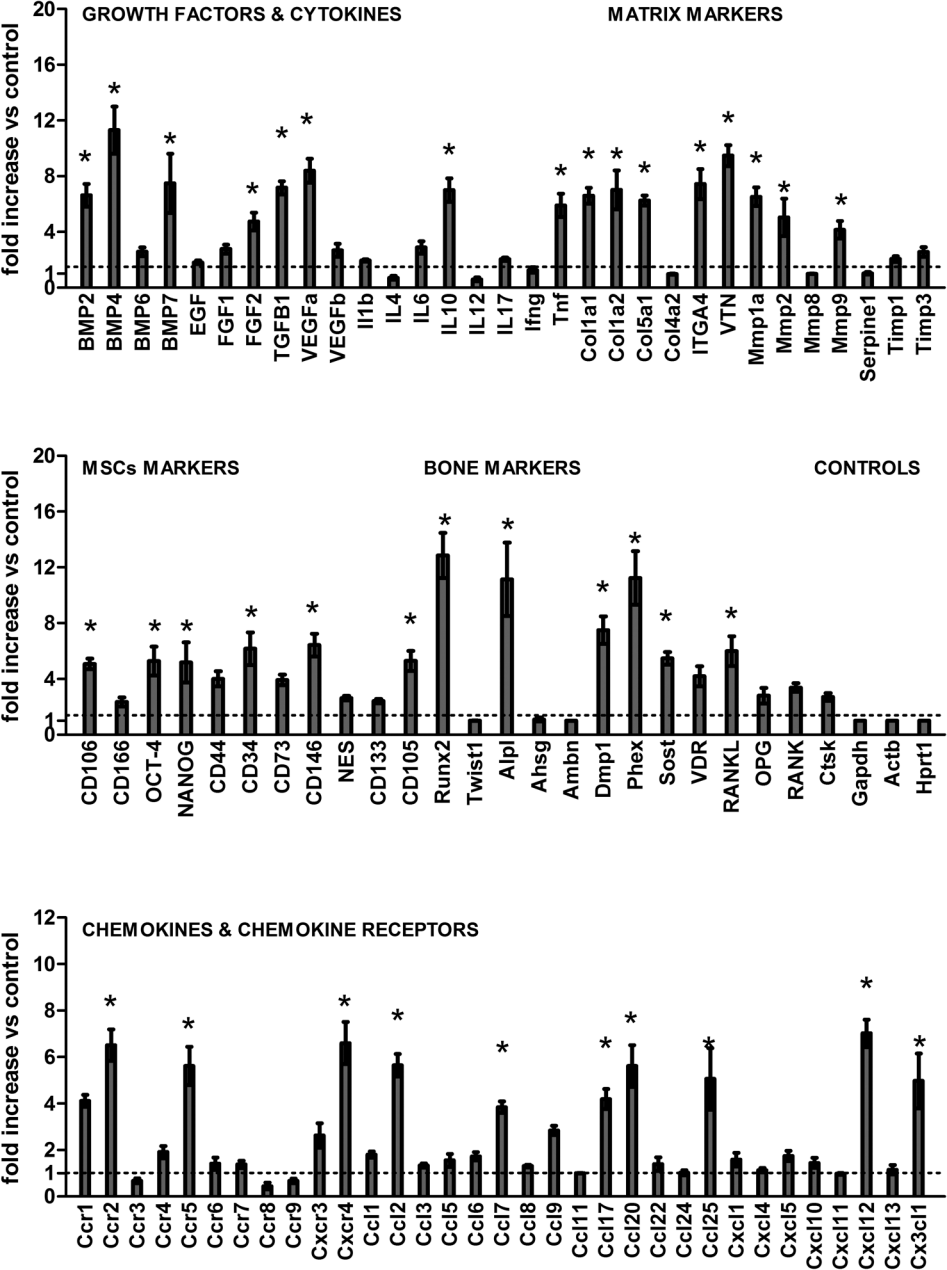
Gene expression patterns in the alveolar bone healing after tooth extraction. Molecular analysis of the gene expression patterns in the bone healing in the socket was comprised of an initial exploratory analysis by RealTimePCR array a pool comprised of samples from all the experimental time periods (0h, 7d, 14d, 21d). RealTimePCR array analysis was performed with the VIA7 system (Applied Biosystems, Warrington, UK) using a customized qPCRarray comprised of the major targets from the Osteogenesis, Inflammatory Cytokines & Receptors and Wound Healing panels of the PCRarrayRT^2^ Profiler (SABiosciences/QIAGEN). Results are depicted as the fold increase change (and the standard deviation) in mRNA expression from triplicate measurements in relation to the control samples and normalized by internal housekeeping genes (GAPDH, HPRT, β-actin). * indicate a statistically significant difference (p<0.05) between the experimental sample and the control.

**Fig 6:**
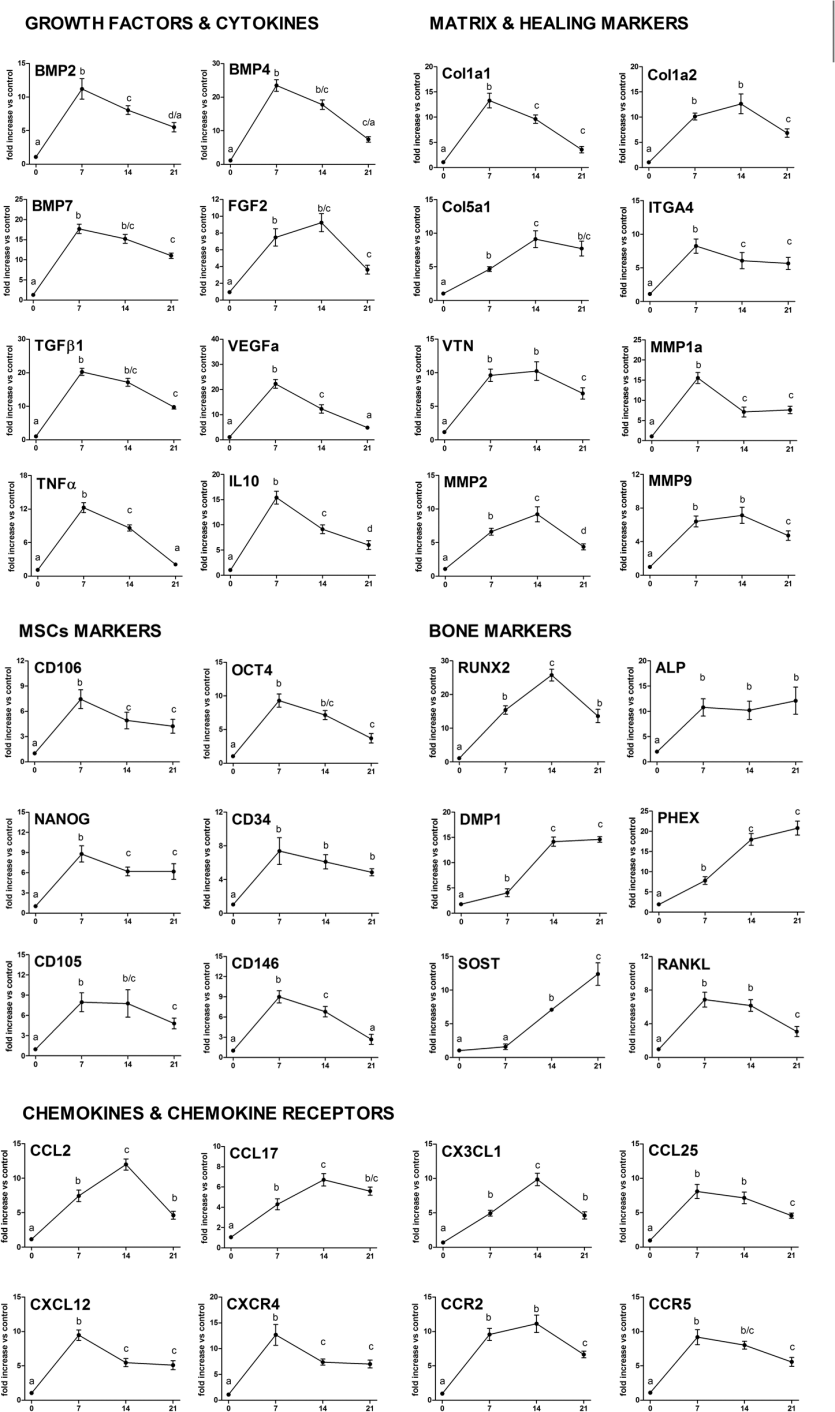
Kinetics of gene expression in the alveolar bone healing process after tooth extraction. After the initial RealTimePCR array pooled from of all the experimental time periods, targets whose expression variation presented a significant variation were analyzed regarding their kinetics expressions in the bone healing process (0h, 7d, 14d, 21d time periods). RealTimePCRarray analysis was performed with the VIA7 system (Applied Biosystems, Warrington, UK) using a customized qPCRarray comprised of the major targets from the Osteogenesis, Inflammatory Cytokines & Receptors and Wound Healing panels of the PCRarrayRT^2^ Profiler (SABiosciences/QIAGEN). Results are depicted as the fold increase change (and the standard deviation) in mRNA expression from triplicate measurements in relation to the control samples and normalized by internal housekeeping genes (GAPDH, HPRT, β-actin). Different letters indicate statistically significant differences (p 0.05) between time periods.

Molecular analysis of the gene expression patterns in the bone healing of the socket was comprised of an initial exploratory analysis by RealTimePCR array (Fig 5), performed with a pool from samples of all experimental time periods, followed by the subsequent analysis of the kinetics of expression of the targets whose expression variation presented a significant variation in the pooled analysis. Among the target molecules, a series of evaluated growth factors, BMP2, BMP4, BMP7, TGFβ1, VEGFa and FGF2 were upregulated throughout the bone healing process in comparison with the control samples (Fig 5). The kinetics analysis (Fig 6) demonstrated that the BMP2, BMP4, BMP7, TGFβ1 and VEGFa expression presented quite similar expression patterns, since they peaked at the 7d time period, followed by a gradual decrease in the mRNA levels. Among the extracellular matrix markers evaluated, the COL1a1, COL1a2, COL5a1, ITGA4, VTN, MMP1a, MMP2 and MMP9 were upregulated in the bone healing process in comparison with the control samples (Fig 5). The kinetics analysis (Fig 6) demonstrated that the COL1a1, ITGA4 and MMP1 peaked at the 7d time period, followed by a gradual decrease in its expression in the subsequent time periods; whereas the VTN remained upregulated at 7 and 14d, while the COL1a2, COL5a1 and MMP2 levels presented a growing curve until 14d time period, followed by a decrease in its mRNA levels. Among the bone markers investigated, the RUNX2, ALPL, DMP1, PHEX, SOST and RANKL were upregulated in the bone healing process in comparison with the control samples (Fig 5). The kinetics analysis (Fig 6) demonstrated that the early bone formation markers RUNX2 and ALPL presented a quick positive regulation, with the ALP detected at higher levels during all the experimental periods, and the RUNX2 expression peaked at 14d, followed by a decrease in its expression. The late bone formation markers DMP1, PHEX and SOST were found to have its mRNA levels upregulated at the 14d and 21d time periods. Among the cytokines and chemokines (and chemokine receptors) investigated, the IL-10, TNFα, CCL2, CCL17, CCL20, CCL25, CXCL12, CX3CL1, CCR2, CCR5 and CXCR4 were positively regulated in the bone healing process in comparison with the control samples (Fig 5). The kinetics analysis (Fig 6) demonstrated that the CXCR4, CXCL12, CCL25, IL-10 and TNFα peaked at the 7d time period, followed by a gradual decrease in the mRNA levels. The CCL2, CCL17 and CX3CL1 levels presented an early upregulation at the 7d time period, followed by an additional increase in its mRNA levels, peaking at the 14d time period and subsequently decreasing. The chemokine receptors CCR2 and CCR5 were positively regulated at the 7 and 14d time periods, and presented a subsequent decrease in its mRNA levels. Mesenchymal stem cells (MSCs) markers expression were identified in the bone healing sites, with the CD106, OCT-4, NANOG, CD34, CD146, CD105 and CXCR4 levels positively regulated in the healing sites when compared with the controls (Fig 5). Kinetics analysis of the MSC markers (Fig 6) demonstrated a significantly higher expression (p <0.05) of these MSCs markers (CD106, OCT-4, NANOG, CD34, CD105, CD146 and CXCR4) at the 7d time period, followed by a gradual decrease in the mRNA levels.

## Discussion

It is well established that the alveolar bone healing is a complex and sequential process involving different cell types and tissues, with a special growing interest in the molecular mechanisms linking the host inflammatory immune response to the effective bone healing [3, 47–49]. Although endochondral bone healing has been extensively studied on fracture healing models, [7, 10, 12] intramembranous bone (such as the alveolar bone supporting the tooth in maxilla and mandible) healing differs from the endochondral bone healing process, and the intramembranous bone healing process remains poorly known, especially in the context of the host response. Therefore, the present study is comprised of a broad (microtomographic, histological, morphometric and molecular) characterization of the intramembranous bone healing process in mice following tooth extraction, which supports its future application for further specific investigations.

Our data demonstrated that following tooth extraction, the initial histological event is comprised of the formation of a blood clot, described as the major initial histological event of the bone healing process in rodents and primates [26]. The formation of a blot clot is described as a fundamental step for the onset of the healing process, since the clot-forming platelets carry a series of growth factors [15]. As the healing process evolves, the blood clot is gradually reabsorbed and replaced by granulation tissue, histologically characterized as a provisional extracellular matrix populated by numerous inflammatory cells, associated with a marked proliferation of fibroblasts and endothelial cells, in accordance with previous histological descriptions [37, 49, 50]. Indeed, PCNA immunostaining confirms the intense cellular proliferation; and the predominance of green fibers in the birefringence analysis endorses the immature nature of the connective matrix, such features being characteristic of the early healing process [51–53]. The development of new blood vessels in the developing granulation tissue is also evident, in agreement with the histological events of alveolar healing previously described in other species [36, 54].

From the molecular point of view, the initial bone healing steps are characterized by expression peaks of growth factors involved in the fibroblasts, osteoblasts and endothelial cells proliferation and differentiation. Molecules such as the vitronectin (VTN) and ITGA4 were also positively regulated, and possibly play a role in cell transmigration, adhesion and spreading, also enhancing the activity of certain growth factors [45, 55]. Growth factors such as the TGFβ1 and FGF-2 can be responsible for promoting the proliferation and activation of fibroblasts, which play an essential role in the synthesis of the provisional matrix of the granulation tissue [56]. In addition, the expression of extracellular matrix components such as the COL1A1 and COL1A2, also considered early bone markers, is abundant in this initial healing stage [38, 57, 58]. The expression of matrix remodeling enzymes is prominent in the initial healing stages. Indeed, MMPs play active roles in the migration of inflammatory cells, degradation and remodeling of extracellular matrix proteins, and in the angiogenesis processes [47, 59–62], described as essential for bone healing [49, 63]. The angiogenic response may be supported by the increased expression of VEGF-A, a major pro-angiogenic factor that acts as a potent stimulator for endothelial cell proliferation [64, 65]. It is also important to highlight the increased expression of pro-inflammatory cytokine TNFα, which also peaks at the 7d time period. TNFα is a classic inflammatory cell migration inducer [66–68], and therefore could account for the leukocytes influx to the granulation tissue during healing. Indeed, TNFα mRNA levels and the inflammatory cells influx peaks match at the 7d time period. Interestingly, the anti-inflammatory IL-10 presents an expression pattern very similar to TNFα, and possibly counteracts the pro-inflammatory response establishing a pro/anti-inflammatory cytokine balance [69].

Subsequently, a reduction of inflammatory infiltrate is accompanied by a gradual replacement of granulation tissue by a mature connective tissue, as previously described in other species [3, 5, 47]. This stage is characterized by an intense activity of fibroblasts synthesizing an extracellular matrix, resulting in the increased content of collagen fibers associated with an additional increase in the density of blood vessels [5, 37]. In accordance, fiber birefringence reveals that the connective tissue maturation results in a decrease in the number of thin and green immature fibers parallel with the accumulation of yellow and red fibers, indicating gradually thicker fibers arranged with a greater degree of compaction [39, 70, 71]. It is important to consider that some of the mature collagen fibers evidenced in the birefringence analysis may be components of developing bone tissue, considered as ‘regular’ ECM constituents. Accordingly, while the Col1a1 and Col1a2 remains positively regulated at the 14d time period, a significant extent of COL1 expression may be derived from cells committed with the osteoblastic phenotype. Accordingly, the expression of COL5a1, produced by differentiated osteoblasts [72], peaks at the 14d time period. In line with this hypothesis, the histomorphometric analysis demonstrated that while the fibroblasts counts significantly decrease in the 7 to 14d interval, the number of osteoblasts remains relatively stable.

Interestingly, while the bone formation at the initial steps of the healing process is modest, peaks of the osteogenic factors expression (such as BMPs) are evidenced. BMP2, BMP4 and BMP7 are central factors in osteoblast differentiation during the physiological and reparative osteogenesis [2, 73, 74], and its expression is possibly the initial trigger for the development of mineralized bone tissue, which takes longer than the development of provisional granulation tissue [21, 75]. In accordance, the expression of RUNX2, the main transcription factor involved in osteoblastic differentiation [20, 38, 76], peaks at 14d, reinforcing the non-immediate nature of osteoblasts differentiation in response to local BMP stimulation. Indeed, the granulation tissue replacement by newly formed bone became evident. The μCT demonstrated that the bone healing occurs as a centripetal process, with the newly formed bone tissue originating from the socket walls and subsequently becoming confluent until filling all the socket extension, in accordance with previous histological descriptions in other animal models [26, 36]. Accordingly, quantitative indicators of bone microarchitectures such as bone volume and trabecular thickness showed a progressive increase over the periods, as well as a marked increase in trabecular number and a reduction in trabecular separation was observed at the 14d time period. The histomorphometric analysis confirms the μCT data, depicting the initial bone formation at the 7d time period, and the gradual increase in the bone mass until the alveolar socket was totally filled by newly formed bone at 21d. Accordingly, a similar kinetics of bone neoformation was previously described in a rat model [3, 5, 77]. The molecular data support the histomorphometric evidences of progressive bone healing at the 14d time period, since markers of advanced osteoblastic differentiation and osteocyte markers, such as PHEX (phosphate-regulating neutral endopeptidase), DMP1 (dentin matrix protein 1) and SOST (sclerostin)[78, 79], are upregulated at this time period.

The extraction socket healing process is considered complete when the dental alveoli are filled by thick bone trabeculae with well-defined medullary canals [80–82], as evidenced at the 21d time period. Once the bone healing is accomplished, these newly formed tissues are subjected to the regular bone remodeling process, which involves a coordinated action of osteoblasts and osteoclasts [3, 5, 37, 47], the increase in the osteoclasts counts being parallel with a stabilization of osteoblasts density observed at this period and is clearly indicative of the ongoing remodeling process [83]. The molecular analysis demonstrated a peak of the key osteoclastogenic factor RANKL at the 7d time period, which possibly accounts for the increased osteoclast counts observed in the subsequent period. As mentioned regarding the osteoblastic differentiation, osteoclast formation is a multi-step process initiated by the fusion of monocytic lineage, which takes a significant amount of time to be completed; as reinforced by the long term *in vitro* culture required to generate osteoclasts, even with proper stimuli [84, 85]. Interestingly, the chemokine receptors CCR2 and CCR5, involved in the chemoattraction of pre-osteoclastic monocytic cells [30, 86, 87], are found to be positively regulated parallel with the periods of high RANKL expression and the peak of osteoclasts counting in the healing site, suggesting that these receptors could account for the osteoclasts formation. In accordance, CCR5 expression was previously described to characterize a F4/80+ cell population, with pro-osteoclastic features in experimental periodontitis [86, 88]. In addition, the blockade of CCR2/CCR5 result in decreased alveolar bone resorption [86, 89], supporting the potential role of these chemokine receptors in the chemoattraction of osteoclast precursors during bone healing. Still in the chemokine/cell migration context, our results demonstrated a significant upregulation of CX3CL1, CCL17, CCL20 and CCL25 in the bone healing. Accordingly, CX3CL1 is described as a pro-reparative chemokine, associated with enhanced myeloid cell and fibroblast recruitment and function, with an angiogenic process [90, 91]. Similarly, CCL17 is described to accelerate wound healing by enhancing fibroblast migration [92]. CCL20 and CCL25 were also previously implicated in the tissue healing in the oral cavity [93]. CCL25 is described as an important mediator of mesenchymal stem cells (MSCs) migration [94], which clearly reinforces it potential pro-reparative role. Since the presence of MSCs is considered essential for tissue repair, CXCR4, a chemokine receptor expressed by bone marrow and periodontal ligament tissue–derived MSCs promotes its mobilization in response to the chemokine CXCL12 [41]. Accordingly, a series of MSCs markers (such as CXCR4, CD106, CD146, OCT-4, NANOG, CD34, CD146, CD105) [41, 95, 96], were upregulated in the bone healing sites, reinforcing the possible participation of MSCs in this process. However, since the cellular phenotypic markers were only evaluated at mRNA level, further confirmatory studies are required to provide a more robust support to the data presented in this study.

**Fig 7:**
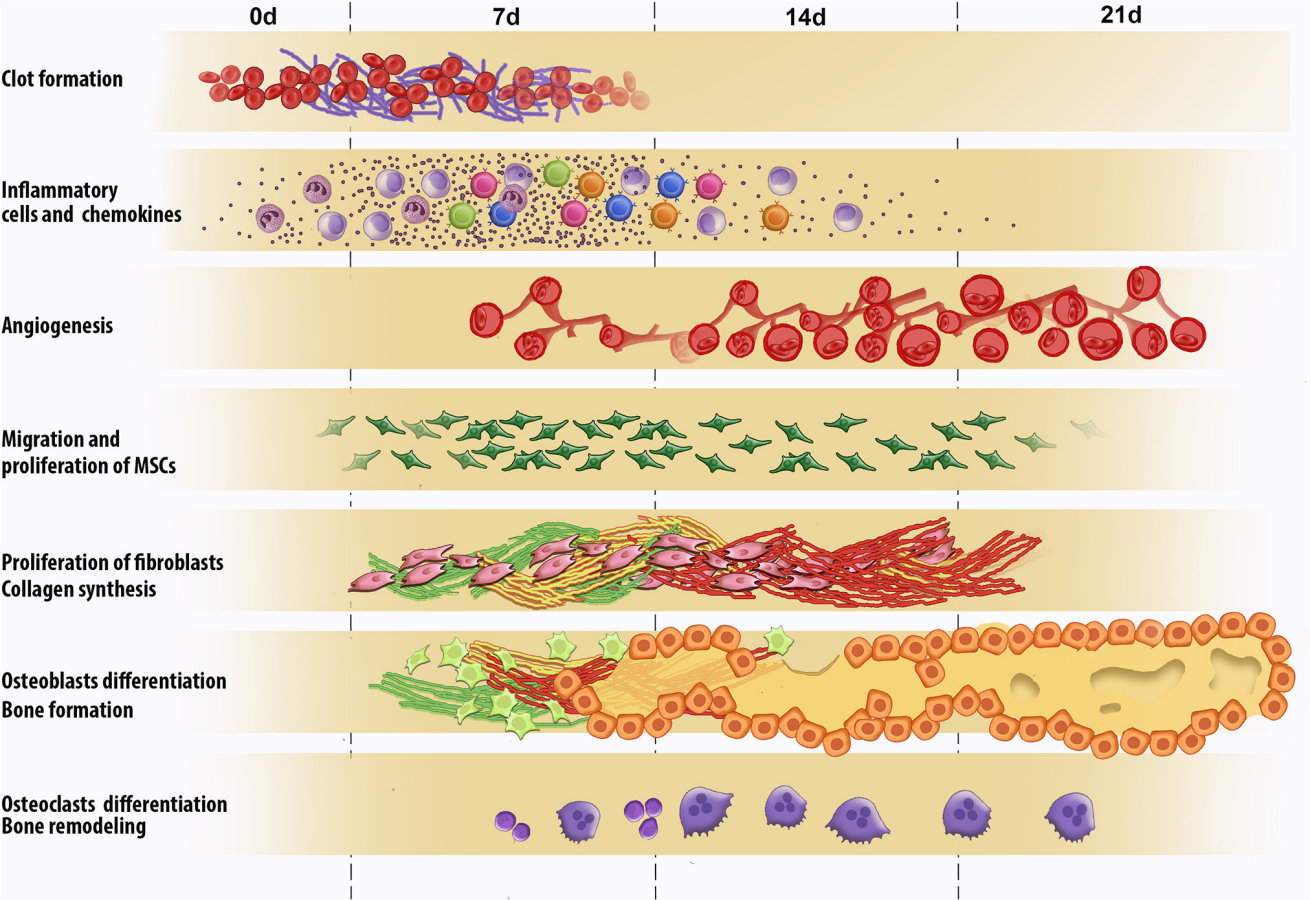
Graphic representation of inflammatory and healing events in the bone healing process after tooth extraction. Briefly, the healing process is sequentially exhibited and at some extent overlapping phases characterized by the presence of clot, migration and proliferation of MSCs, granulation tissue formation with inflammatory infiltrate, angiogenesis, proliferation of fibroblasts and collagen synthesis. And finally bone formation followed by subsequent remodeling.

Thus, it is possible to affirm that the histological feature of alveolar healing observed in our study in mice showed a response pattern very similar to other animal models described in the literature, as schematically summarized in the Fig 7.

Taking into account the chronology well established in different species [97], the kinetics of some histological events, really resemble the steps of alveolar healing previously described in rats [5, 38, 98], dogs [37, 99], monkeys [36], and even humans [100]. Importantly in this study, no evidences of cartilage cells or tissue were observed, strengthening the intramembranous nature of bone healing. However, despite the histological evidences towards the absence of cartilage along the bone repair, since the expression of specific markers of cartilage formation such as COL2A1, COL9A1 and COL11A1 was not evaluated, it is not possible to rule out that such molecules were expressed, even in a transient way [101, 102], during the bone repair process. The birefringence and μCT analysis support the histological findings, and the molecular analysis of the broad panel of growth factors, cytokines, chemokines, matrix and healing markers, as well the MSCs and bone cell markers, provide a novel and interesting parallel between the morphological events and the underlying mechanisms that couple the host inflammatory immune response with successful alveolar bone healing. In conclusion, based on the set of results presented here, we can suggest that this experimental model in mice may contribute to understanding the underlying mechanisms of the intramembranous bone healing process.

## Conclusion

In summary, the present study describes a mouse model of intramembranous bone healing, which resembles the morphological aspects of alveolar bone healing previously described in other animal models and in humans. While additional and novel birefringence and μCT analysis support the histological findings, the broad molecular analysis performed demonstrated the differential expression of several growth factors, cytokines, chemokines, matrix and healing markers, as well MSCs and bone cells markers, in the bone healing process, providing an original parallel between the morphological events and the underlying mechanisms, with a special focus in host inflammatory immune response related events, which probably account for the successful alveolar bone healing. Finally, due to the extensive knowledge regarding mice inflammatory and immunological responses and the existence of multiple strains and experimental reagents, the characterization of mice as a suitable model for intramembranous bone healing opens a wide window of possibilities for further experimentation.

## Supporting Information

S1 Fig Experimental protocol of a murine tooth extraction model. A. Extraction of the upper right incisor in C57BL/6 wild-type (WT) mice using a stereomicroscope and dental exploratory probe. B. Clinical tweezers for seizing and removal of the tooth. C. Socket after tooth extraction. D. Size of the upper right incisor removed.
(TIF)

S2 Fig Immunohistochemistry for the proliferating cell nuclear antigen (PCNA) along the bone healing process. A. Representative section of the alveolar healing process at 7, 14 and 21 days post tooth extraction showed positive immunostaining for Proliferating Cell Nuclear Antigen (PCNA). Quantitative analysis of immunolabeled cells was performed similar to that described in the histomorphometric analysis, and the results are presented as the density (±SEM) of PCNA+ cells (B) and total number (±SEM) of PCNA+ cells (C). Different letters indicate statistically significant differences (p 0.05) between time periods. Immunohistochemistry methods: histological sections were deparaffinized following standard procedures. The material was pre-incubated with a 3% hydrogen peroxidase blocker (Spring Bioscience Corporation, CA, USA) and subsequently incubated with 7% non-fat dry milk to block the serum proteins. The slices were then incubated with anti-PCNA polyclonal primary antibodies (sc-9857) from Santa Cruz Biotechnology, (Santa Cruz, CA, USA), at 1:100 concentrations for 1h at room temperature. A universal immuno-enzyme polymer method was used and sections were incubated in immunohistochemical staining reagent for 30min at room temperature. Visualization of the antigen–antibody reaction was performed using DAB and counterstaining with Mayer's hematoxylin. For control studies of the antibodies, some serial sections were treated only with the Universal immuno-enzyme polymer, in a separate preparation. Positive controls were performed in the mouse testis for PCNA. The determination of immunolabeling cells for each antibody was performed quantitatively as described for the histomorphometric analysis.
(TIF)
