# Supplementary figures and images for: Intramembranous Bone Healing Process Subsequent to Tooth Extraction in Mice: Micro-Computed Tomography, Histomorphometric and Molecular Characterization
Source: PLoS One. 2015 May 29;10(5):e0128021. doi: 10.1371/journal.pone.0128021 (PMC4449187; doi:10.1371/journal.pone.0128021)

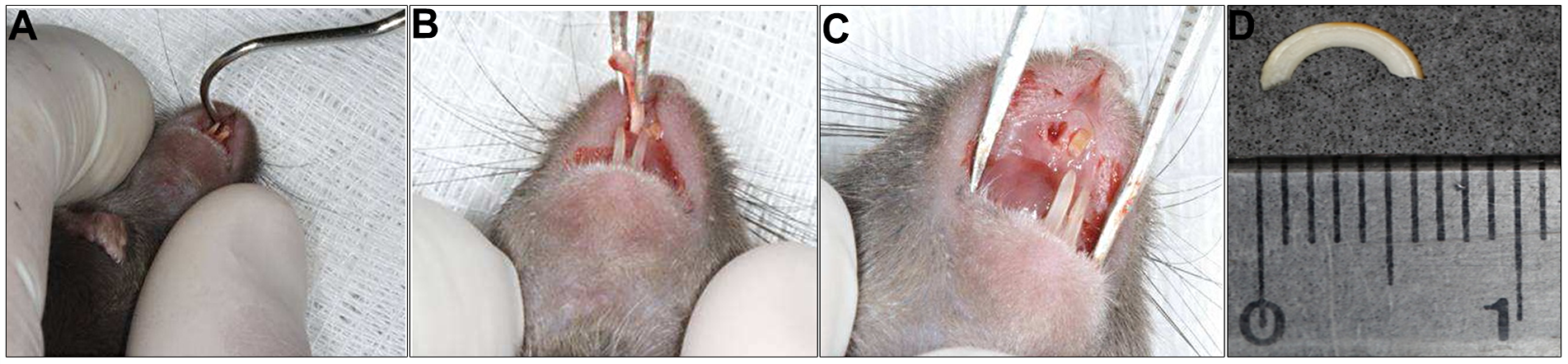

Supplement: S1 Fig — A. Extraction of the upper right incisor in C57BL/6 wild-type (WT) mice using a stereomicroscope and dental exploratory probe. B. Clinical tweezers for seizing and removal of the tooth. C. Socket after tooth extraction. D. Size of the upper right incisor removed. (TIF) [file pone.0128021.s001.tif]

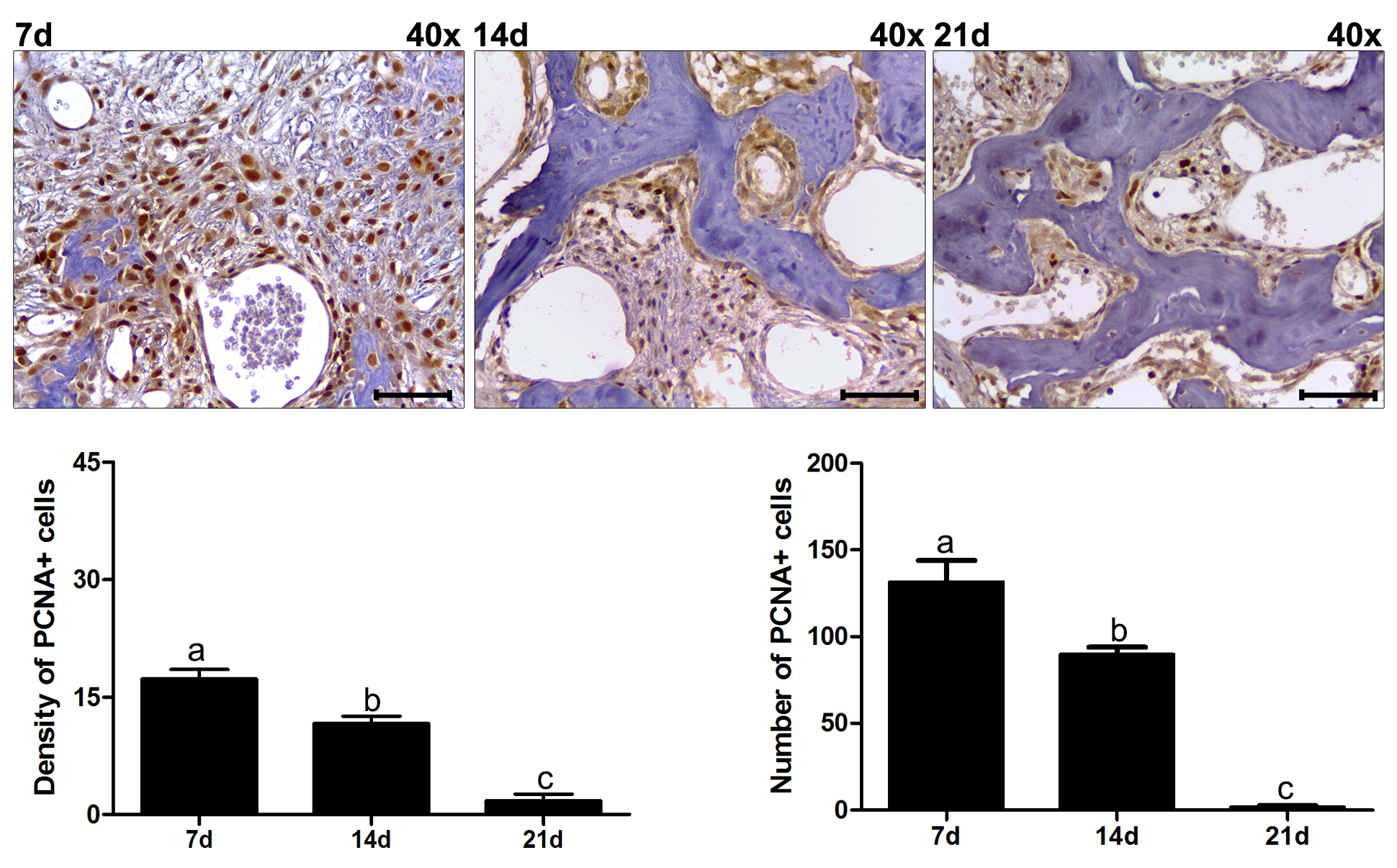

Supplement: S2 Fig — A. Representative section of the alveolar healing process at 7, 14 and 21 days post tooth extraction showed positive immunostaining for Proliferating Cell Nuclear Antigen (PCNA). Quantitative analysis of immunolabeled cells was performed similar to that described in the histomorphometric analysis, and the results are presented as the density (±SEM) of PCNA+ cells (B) and total number (±SEM) of PCNA+ cells (C). Different letters indicate statistically significant differences (p 0.05) between time periods. Immunohistochemistry methods: histological sections were deparaffinized following standard procedures. The material was pre-incubated with a 3% hydrogen peroxidase blocker (Spring Bioscience Corporation, CA, USA) and subsequently incubated with 7% non-fat dry milk to block the serum proteins. The slices were then incubated with anti-PCNA polyclonal primary antibodies (sc-9857) from Santa Cruz Biotechnology, (Santa Cruz, CA, USA), at 1:100 concentrations for 1h at room temperature. A universal immuno-enzyme polymer method was used and sections were incubated in immunohistochemical staining reagent for 30min at room temperature. Visualization of the antigen–antibody reaction was performed using DAB and counterstaining with Mayer's hematoxylin. For control studies of the antibodies, some serial sections were treated only with the Universal immuno-enzyme polymer, in a separate preparation. Positive controls were performed in the mouse testis for PCNA. The determination of immunolabeling cells for each antibody was performed quantitatively as described for the histomorphometric analysis. (TIF) [file pone.0128021.s002.tif]
